# Fecal microbiota associated with phytohaemagglutinin‐induced immune response in nestlings of a passerine bird

**DOI:** 10.1002/ece3.4454

**Published:** 2018-09-20

**Authors:** Jakub Kreisinger, Lucie Schmiedová, Adéla Petrželková, Oldřich Tomášek, Marie Adámková, Romana Michálková, Jean‐François Martin, Tomáš Albrecht

**Affiliations:** ^1^ Department of Zoology Faculty of Science Charles University Prague Czech Republic; ^2^ Department of Ecology Faculty of Science Charles University Prague Czech Republic; ^3^ Czech Academy of Sciences Institute of Vertebrate Biology Brno Czech Republic; ^4^ Montpellier‐SupAgro UMR CBGP Montferrier‐sur‐Lez France

**Keywords:** fitness, immunity, inflammation, metabarcoding, microbiome, symbiosis

## Abstract

The vertebrate gastrointestinal tract is inhabited by a diverse community of bacteria, the so‐called gut microbiota (GM). Research on captive mammalian models has revealed tight mutual interactions between immune functions and GM. However, our knowledge of GM versus immune system interactions in wild populations and nonmammalian species remains poor. Here, we focus on the association between GM community structure and immune response measured via the phytohaemagglutinin (PHA) skin swelling test in 12‐day‐old nestlings of a passerine bird, the barn swallow (*Hirundo rustica*). The PHA test, a widely used method in field ecoimmunology, assesses cell‐mediated immunity. GM structure was inferred based on high‐throughput 16S rRNA sequencing of microbial communities in fecal samples. We did not find any association between PHA response and GM diversity; however, our data revealed that the intensity of PHA response was correlated with differences in GM composition at the whole‐community level. Ten bacterial operational taxonomic units corresponding to both putative commensal and pathogens were identified as drivers of the compositional variation. In conclusion, our study suggests existence of GM versus immune system interactions in a free‐living nonmammalian species, which corresponds with previous research on captive vertebrates.

## 1 INTRODUCTION

Vertebrates harbor diverse microbial communities in their guts (Ley et al., 2008; Qin et al., 2010) and these so‐called gut microbiota (GM) are involved in many interactions with the host. In addition to its effect on gut function (Jumpertz et al., 2011; Sekirov, Russell, Antunes, & Finlay, 2010), interactions with the host's immune system have important consequences for the host's health and fitness. The different species comprising the GM regulate the host immune system contribute to its development during early ontogenetic stages (Belkaid & Hand, 2014; Kim, Park, & Kim, 2014; Sjögren et al., 2009; Wu & Wu, 2012) and affect the host's capacity to resist invading pathogens (Ivanov et al., 2009). Simultaneously, the host supports a wide range of mechanisms, usually linked with the immune genes, that regulate GM content (Benson et al., 2010; Bolnick et al., 2014).

Most current research focussed on interactions between GM, and the host immune system has used captive‐bred animals as a model. However, both taxonomic and functional composition varies considerably between wild and captive populations (Kreisinger, Čížková, Vohánka, & Piálek, 2014; McKenzie et al., 2017). Similarly, both immune parameters and their interindividual variation differ between wild and captive populations due to the altered genetic background of laboratory strains, a lower prevalence of parasites and pathogens and less variation in biotic and abiotic factors involved in immune trait modulation under captive conditions (Boysen, Eide, & Storset, 2011; Flies, Mansfield, Grant, Weldele, & Holekamp, 2015). Hence, results for GM versus immune system interactions obtained from captive populations do not necessarily reflect the selective forces that shape the host's immune system over GM‐associated coevolutionary history (Maizels & Nussey, 2013). Moreover, our knowledge on host immune system versus GM interactions is largely based on mammalian species hosting different GM and having distinct immune system than other vertebrate taxa. Specifically, bacteria from the Firmicutes and Bacteroidetes phyla typically dominate in the mammalian GM (Ley et al., 2008), whereas nonmammalian vertebrate GM may comprise taxonomically more diverse bacterial consortia (Kropáčková, Těšický, et al., 2017; Sullam et al., 2012). Consequently, further studies dealing with free‐living, nonmammalian species are essential for a deeper understanding of the evolutionary forces shaping interactions between GM and the host's immune system.

Here, we study the associations between GM structure and immune response in nestlings of a free‐living passerine bird, the barn swallow (*Hirundo rustica*). The barn swallow is a migratory, insectivorous species with complex social system that breeds in colonies (Cramp & Perrins, 1993; Petrželková et al., 2015). The GM of barn swallows and other birds differs from that of conventional mammalian models (Hird, Carstens, Cardiff, Dittmann, & Brumfield, 2014; Kropáčková, Těšický, et al., 2017; Waite & Taylor, 2014), which makes birds a valuable model group for gaining a deeper insight into GM versus immune system interactions. Various aspects of immune system function have previously been studied in barn swallows and other free‐living birds, predominantly related to reproductive behavior and sexual selection (Møller, 2001; Saino, Ambrosini, Martinelli, & Møller, 2002; Saino, Ferrari, Romano, Martinelli, & Møller, 2003). However, there have been few studies aimed at testing the association between immunity and associated microbial communities (Ruiz‐Rodríguez et al., 2009).

We analyzed fecal microbiota profiles using high‐throughput sequencing of 16S rRNA amplicons as a proxy for GM. Immune response was assessed via the phytohaemagglutinin (PHA) skin swelling test, which is the most widely used method for assessment of cell‐mediated response in field ecoimmunology (Møller, 2001; Saino et al., 2002, 2003; Tella, Lemus, Carrete, & Blanco, 2008). The PHA assay is traditionally believed to reflect adaptive immune response mediated predominantly by T cells (Goto, Kodama, Okada, & Fujimoto, 1978; Tella et al., 2008). However, recent research suggests that immune mechanisms involved in PHA‐induced swelling are more complex, comprising a strong component of innate immunity (Vinkler, Bainová, & Albrecht, 2010; Vinkler, Schnitzer, Munclinger, & Albrecht, 2012). A stronger PHA response is typically interpreted as beneficial due to its positive association with fitness‐ and condition‐related traits (Bowers et al., 2014). Given the complex immunological background of PHA swelling, however, this may not hold universally. There are numerous examples showing no, or even a negative, relationship between PHA responsiveness and body condition, physiological stress, or health status (Møller & Petrie, 2002; Saks, Karu, Ots, & Hõrak, 2006; Vinkler et al., 2012). Despite these complexities, it is worth exploring the potential correlations between GM and PHA responsiveness as PHA‐induced swelling is the most widely studied trait in ecoimmunological literature. In addition, previous research supports both positive (Saks et al., 2006) and negative (Merlo, Cutrera, & Zenuto, 2016) association between gut infection by eukaryotic parasites and PHA responsiveness, suggesting that extending such research on prokaryotic communities inhabiting the gut could be potentially fruitful. We therefore combine data on GM profiles with measures of PHA swelling in order to test whether there is any association between GM diversity and immune response in barn swallows. We also assess whether interindividual variation in PHA response is correlated with differences in GM composition and which specific bacterial taxa determine any such variation in PHA response.

## 2 METHODS

### 2.1 Field data acquisition

Data on fecal microbiota and PHA response were collected during 2014 (late April – late June) from barn swallow nestlings (*n* = 58) distributed in 32 clutches (Czech Republic, 49° 4′ 7.762″ N, 14° 42′ 36.521″ E, Supporting Information Table S1).

Tissue thickness of the left wing web (patagium) of 12‐day‐old barn swallow nestlings was measured using a standard thickness gauge (Mitutoyo, Japan). Subsequently, the PHA solution (0.10 mg of PHA‐P dissolved in 20 μl of DPBS) was injected and the magnitude of the swelling reaction was measured after 24 hr. Both pre‐ and posttreatment tissue thickness measurements were performed three times by the same person (A.P., accuracy ~0.01 mm). Repeatability of these measurements was high (intraclass correlation coefficient = 0.973 and 0.967 for pre‐ and posttreatment measurements, respectively). Consequently, the average tissue thickness increment between pre‐ and posttreatment measurements was used as an index of PHA‐induced swelling in subsequent analyses.

Fecal samples of 12‐day‐old barn swallow nestlings were collected prior PHA injection, placed in sterile cryotubes (Simport, Canada), and stored in liquid nitrogen during field works. After the field works, samples were preserved under −80°C until DNA extractions. Further details on fecal sample collection and storage, together with a description of breeding site, are provided elsewhere (Kreisinger et al., 2017).

All field procedures were conducted in accordance with the Guidelines for Animal Care and Treatment of the European Union and approved by the Animal Care and Use Committees of the Czech Academy of Sciences (041/2011) and Charles University (4789/2008‐0).

### 2.2 Microbiome profiling and bioinformatic processing of 16S rRNA data

Metagenomic DNA was isolated from fecal samples using PowerSoil Mo Bio kits (Qiagen). The V3‐V4 region of 16S rRNA was amplified using S‐D‐Bact‐0341‐b‐S‐17 (CCTACGGGNGGCWGCAG) and S‐D‐Bact‐0785‐a‐A‐21 (GACTACHVGGGTATCTAATCC) primers (Klindworth et al., 2013), tagged with 10 bp oligonucleotide indices for demultiplexing. Technical PCR duplicates were prepared for all samples in order to check for microbial profile consistency. Sequencing libraries were prepared using TruSeq Nano Kits and sequenced on Illumina Miseq using v3 chemistry.

The resulting 300 bp long paired‐end reads were merged using Pear (Zhang, Kobert, Flouri, & Stamatakis, 2014) and demultiplexed using Mothur (Schloss et al., 2009). Lotus pipeline (Hildebrand, Tadeo, Voigt, Bork, & Raes, 2014) was used for quality filtering (elimination of sequences, if average *Q* < 30 and if average *Q* within 50 bp sliding dropped below 25) and elimination of chimeric sequences. Subsequently, UPARSE algorithm (Edgar, 2013) implemented in Lotus was used for clustering of resulting high‐quality reads at 97% similarity threshold to operational taxonomic units (OTUs). Taxonomic assignment of representative sequences for each OTU was performed using RDP classifier and Green Genes database (v. 13_5, DeSantis et al., 2006) as a reference. Representative sequences were aligned using PyNAST (Caporaso et al., 2010) and a phylogenetic tree constructed using FastTree (Price, Dehal, & Arkin, 2010). The OTU table, sample metadata, taxonomic annotations, and phylogenetic tree were stored as a phyloseq object (McMurdie & Holmes, 2013) for further analysis. OTUs not assigned to phylum level, or those classified as chloroplasts (1% and 8.2% reads, respectively), were considered as sequencing artefacts and diet contaminants, respectively, and eliminated from all downstream analyses. Details on laboratory procedures associated with microbiome profiling and bioinformatic processing of sequencing data were provided in a previous study on this species (Kreisinger et al., 2017).

### 2.3 Statistical analysis

Barn swallow GM taxonomic content was visually summarized using Krona tools (Ondov, Bergman, & Phillippy, 2011). All the statistical analyses were conducted using packages running under R 3.4.3 software (R Core Team, 2016). As we detected significant correlation between PHA response and Julian date of fecal sample collection (Pearson *r* = 0.324, *p* < 0.01), we controlled all subsequent statistical analyses for effect of sampling date. Association between microbiota diversity (i.e., number of observed OTUs, Chao1 diversity estimates and Shannon diversities) and PHA response or Julian date of sample collection was tested using linear mixed‐effect models (LME, R package lme4; Bates, Mächler, Bolker, & Walker, 2015) with Gaussian distribution of errors. Nest identity was included as a random intercept. Next, weighted UniFrac (Lozupone & Knight, 2005) and Bray–Curtis community dissimilarity between samples were calculated based on sample‐specific OTU proportions. The effect of PHA‐induced response and Julian date was assessed using distance‐based redundancy analysis (db‐RDA, Legendre & Anderson, 1999), with the matrix of between‐sample dissimilarities included as a response. Permutation‐based ANOVA (anova.cca function from R package vegan, Oksanen et al., 2013) was then used to test for significance of the constrained db‐RDA axes. According to these analyses, only the first constrained db‐RDA axis was significant (*p* < 0.001 for both UniFrac and Bray–Curtis dissimilarity), and the effect of the second constrained db‐RDA axis was nonsignificant (*p* > 0.3 in both cases). We then extracted the scores for the first db‐RDA axis and tested whether they were significantly associated with PHA response and/or Julian date using LME. We argue that this analysis method is preferable to the default anova.cca, as this function cannot effectively account for pseudoreplications induced by sampling multiple individuals from the same nest.

Associations between abundance of OTUs and PHA response were tested using generalized LMEs from R package BhGLM for data with negative binomial distribution of errors (Zhang et al., 2017). OTU‐specific read counts within individual samples were included as a response, while Julian date of sample collection and PHA response were included as explanatory variables. Log‐transformed total number of reads per sample was specified as model offset (i.e., assuming number of reads per given OTU to be proportional to total number of reads per individual sample) and clutch identity as random effect. The qvalue method (Storey & Tibshirani, 2003) was subsequently used to account for false discoveries due to multiple testing. To optimize sensitivity of OTU‐level analyses, we applied “independent filtering” procedure (Bourgon, Gentleman, & Huber, 2010) using DESeq2 R package (Love, Huber, & Anders, 2014) and considered only OTUs that passed this step (*n* = 196 OTUs, representing 96% of all high‐quality reads). Procrustean analysis revealed high congruence between original and subsetted microbial profiles (Procrustean *r* = 0.9974, Procrustean sum of squares = 0.0052, *p* < 0.0001), suggesting that resulting OTU subset covered representative variation in the GM content.

## 3 RESULTS AND DISCUSSION

**Figure 1:**
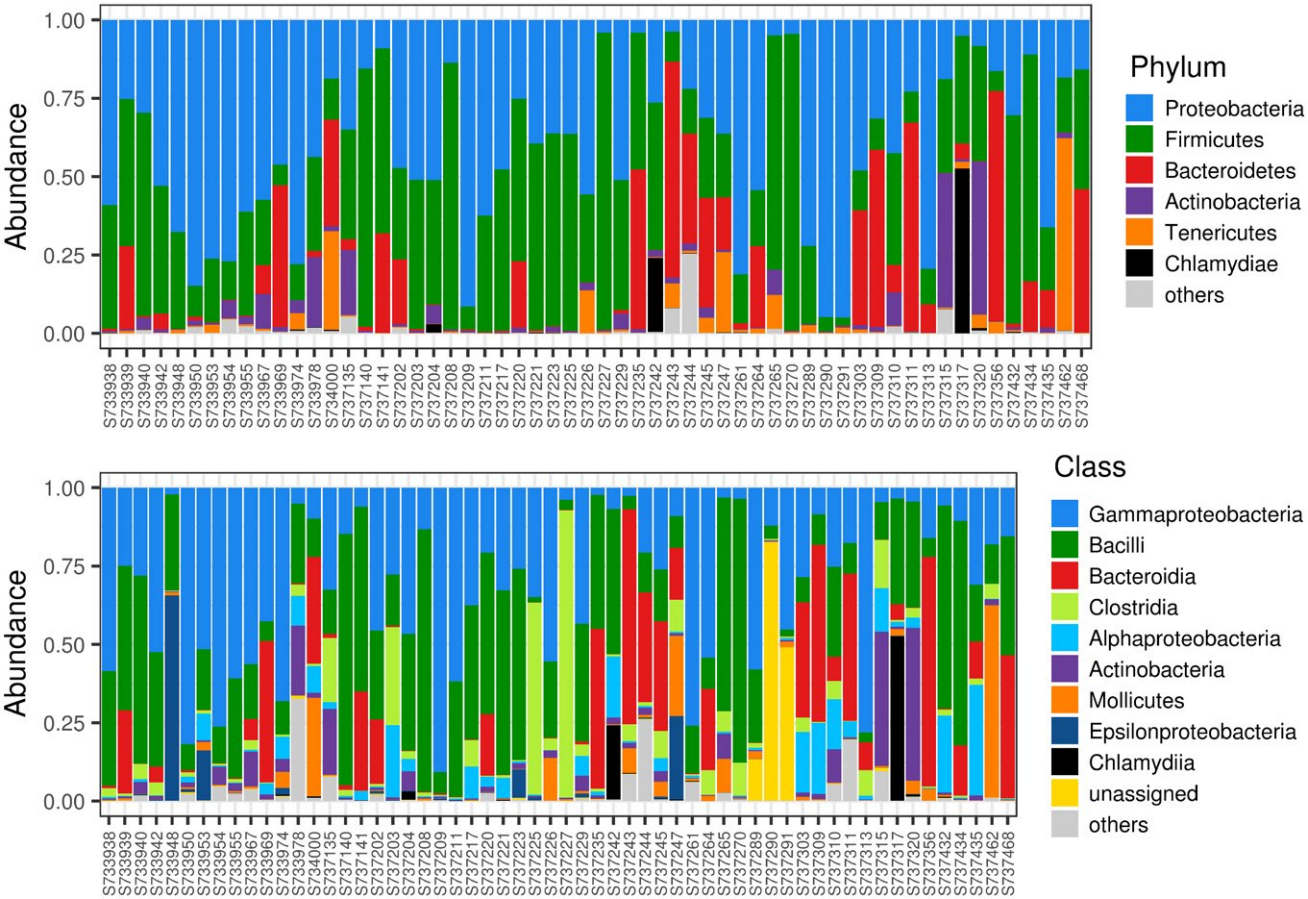
Bar plots indicating proportions of dominant bacterial phyla and classes in barn swallow fecal microbiota samples

**Figure 2:**
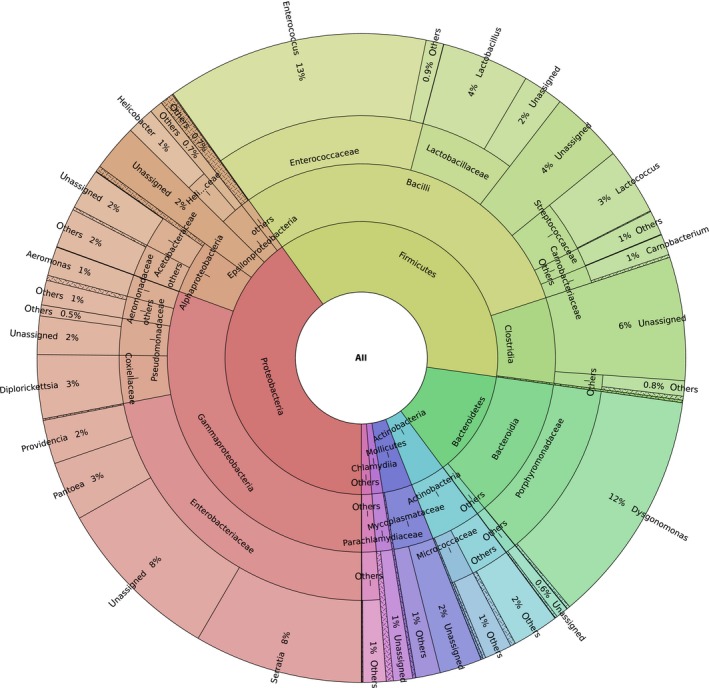
Summary of barn swallow GM taxonomic content. Rare taxa (represented by <2% reads) are labeled as “others”

After all filtering steps, we obtained 947,675 high‐quality reads with a median sequencing depth per sample of 13,798 (range = 1,112–44,777) and an average number of 97% UPARSE OTUs per sample of 153.6 (range = 67–443). In line with our previous study on barn swallow nestlings from the same population (Kreisinger et al., 2017), the most abundant bacterial phyla were Proteobacteria (dominated by *Serratia*,* Pantoea*,* Providencia,* and *Diplorickettsia*) Firmicutes (dominated by genera *Enterococcus*,* Lactococcus*,* Lactobacillus,* and unassigned *Clostridia*), Bacteriodetes (dominated by the genus *Dysgonomonas*), and Actinobacteria. All Actinobacterial genera were represented by low percentage of reads (<1%), with *Rhodococcus* (0.58% of all read), *Rothia* (0.56% of reads), and *Corynebacterium* (0.55% of reads) being the most abundant (Figures 1 and 2).

We did not observe any association between PHA response and GM diversity (LME: *p* > 0.2 for all alpha diversity measures; Table 1). In theory, a correlation between immune functions and GM diversity might be expected as mutual interactions between immune gene allelic diversity (Bolnick et al., 2014), intensity of immune response (Hawley, Sydenstricker, Kollias, & Dhondt, 2005), parasite load (Kurtz et al., 2004; Madsen & Ujvari, 2006; Sommer, 2005), and overall GM diversity have repeatedly been reported in previous studies. However, consistent with our data, number of previous studies did not find any straightforward correlation between GM richness and immune phenotype (Chang, Hao, Offermanns, & Medzhitov, 2014; Jones et al., 2013; Vatanen et al., 2016).

**Table 1:** Effect of PHA response and Julian date on GM alpha diversity (assessed as number of observed OTUs, Chao1 predictions of total GM diversity and Shannon index). Shown are LME estimates (Estimate) and corresponding standard errors (*SE*), deviance changes due to elimination of a given term from the model (*χ* ^2^) and associated degrees of freedom (Δ*df*), and probability values (*p*)

Response	Explanatory var.	Estimate	*SE*	*χ* ^2^	Δ*df*	*p*
Chao1	Intercept	1.897	0.079			
	Julian	−0.001	0.001	3.364	1	0.067
	PHA	0.028	0.024	1.388	1	0.239
Observed	Intercept	1.780	0.096			
	Julian	−0.001	0.001	0.933	1	0.334
	PHA	0.026	0.030	1.149	1	0.284
Shannon	Intercept	0.600	0.685			
	Julian	0.000	0.005	0.002	1	0.968
	PHA	0.152	0.208	0.645	1	0.422

*Note*

GM: gut microbiota; LME: linear mixed‐effect model; OTUs: operational taxonomic units; PHA: phytohaemagglutinin.

**Figure 3:**
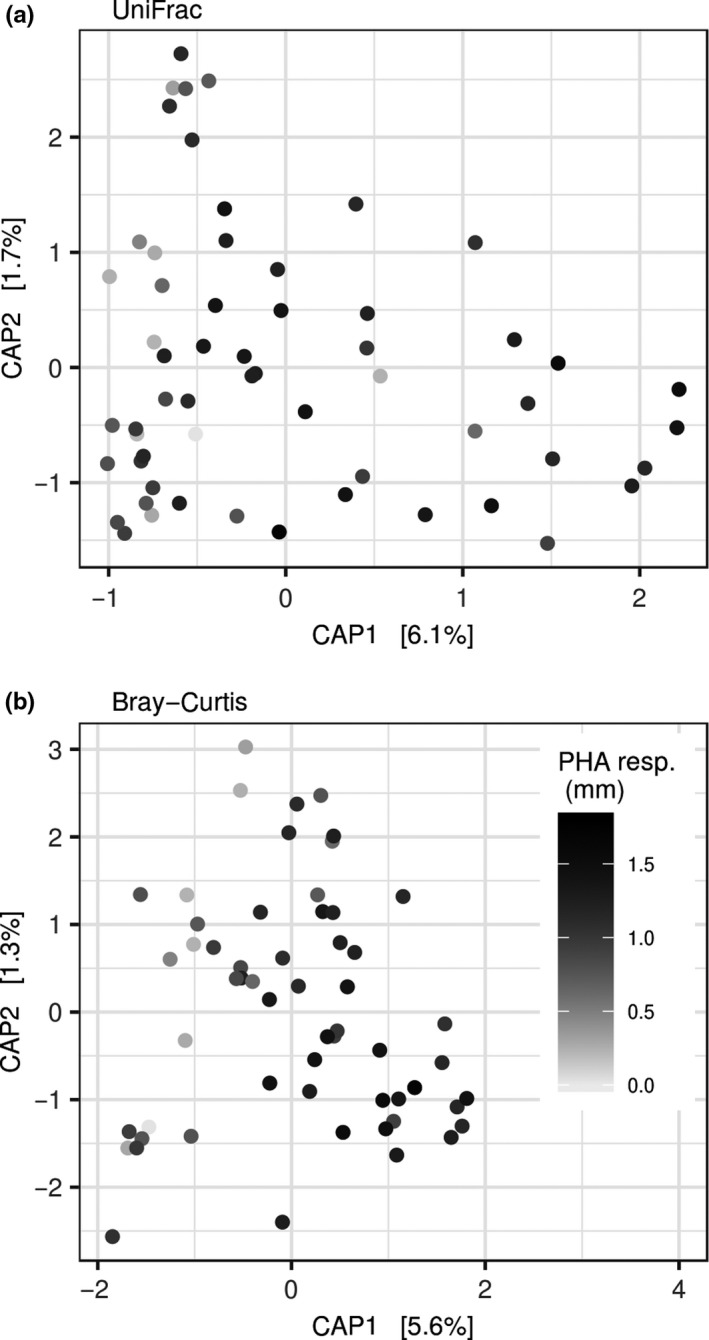
Db‐RDA ordination of GM in barn swallow nestlings. Two dissimilarity types between samples were used as a response (Bray–Curtis and weighted UniFrac), while PHA swelling and Julian date of sampling were included as explanatory variables. Variation along the first two constrained axes is shown. Strength of PHA response (in millimeters) is indicated by color intensity of plotting characters

Despite the lack of any relationship between GM diversity and PHA response, we observed significant correlation between magnitude of PHA swelling and variation in GM composition at the whole‐community level, suggesting that individuals with similar GM composition had a similar PHA response. This association was specifically implied based on db‐RDA ordination (Figure 3). In addition, LMEs running on scores for the first db‐RDA axis (using both Bray–Curtis and weighted UniFrac distance) revealed a significant effect of PHA response after statistical control for Julian date of sample collection, while the effect of Julian date itself was significant only in the case of db‐RDA for Bray–Curtis dissimilarity (Table 2).

**Table 2:** Effect of PHA response and Julian date on GM composition (corresponding to the first db‐RDA axis for weighted UniFrac and Bray–Curtis dissimilarity). Shown are LME estimates (Estimate) and corresponding standard errors (*SE*), deviance changes due to elimination of a given term from the model (*χ* ^2^) and associated degrees of freedom (Δ*df*), and probability values (*p*)

Response	Explanatory var.	Estimate	*SE*	*χ* ^2^	Δ*df*	*p*
Bray–Curtis	Intercept	−5.053	0.953			
	Julian	0.024	0.006	13.479	1	<0.001
	PHA	0.911	0.294	8.724	1	0.003
UniFrac	Intercept	−2.074	1.262			
	Julian	0.007	0.008	0.871	1	0.351
	PHA	0.769	0.366	4.523	1	0.033

*Note*

GM: gut microbiota; LME: linear mixed‐effect model; PHA: phytohaemagglutinin.

According to OTU‐centered negative binomial LMEs, ten OTUs represented by relatively low number of reads (~2.2% in total) exhibited significant association with the intensity of PHA response (Table 3 and Supporting Information File S1). Two of these OTUs, belonging to Lactic Acid Bacteria from genus *Enterococcus* and *Lactococcus,* were negatively related to PHA swelling. Intensity of PHA‐induced swelling seems to strongly reflect general proinflammatory potential of given individual (Vinkler et al., 2010). Consequently, observed negative correlation between *Lactococcus* abundances and PHA response can be related to anti‐inflammatory effect that was previously described for some probiotic species from this genus (Han, Lee, Park, & Paik, 2015; Luerce et al., 2014). There are several plausible explanations for the negative correlation between abundances of *Enterococcus* OTU and PHA response. Similarly, as in the case of *Lactococcus*, some *Enterococcus* species exhibit probiotic properties. However, *Enterococcus* genus includes also several pathogenic strains, whose infection can directly affect host's immunity (Fisher & Phillips, 2009). Unfortunately, we are not able to distinguish between these two alternatives as 16S rRNA region used in our study does not allow reliable species‐level assignment of this particular OTU. Interestingly, association of *Enterococcus* loads and phenotype was observed in another study on passerine juveniles (González‐Braojos, Vela, Ruiz‐de‐Castañeda, Briones, & Moreno, 2012). In particular, *Enterococcus* loads were negatively correlated with growth rates, that is, the phenotype trait that can covary with PHA response (Lifjeld, Dunn, & Whittingham, 2002) and other immune parameters as well (van der Most, de Jong, Parmentier, & Verhulst, 2011). Reduced PHA response was also associated higher abundances of OTU from genus *Rickettsia*, an insect‐borne intracellular pathogen (Parola & Didier, 2001) commonly detected bird GM (Kropáčková, Těšický, et al., 2017).

**Table 3:** OTUs associated with PHA response according to generalized LMEs for data with negative binomial errors. Shown are taxonomic assignation of individual OTUs, LME‐based estimates, and corresponding standard errors (*SE*) for OTU abundance versus PHA response association, together with their probability (*p*) and *q* values

OTU name	Estimate	*SE*	*p* Value	*q* Value	Class	Family	Genus
OTU_105	−5.7648	1.0746	<0.001	0.0005	Lactobacillales	Streptococcaceae	*Lactococcus*
OTU_446	−5.5817	1.2421	0.0001	0.0035	Clostridiales	Unassigned	Unassigned
OTU_77	−3.774	0.9505	0.0005	0.0111	Lactobacillales	Enterococcaceae	*Enterococcus*
OTU_155	−3.5573	0.989	0.0013	0.0207	Pseudomonadales	Pseudomonadaceae	Unassigned
OTU_50	−2.6995	0.7293	0.001	0.0186	Rickettsiales	Rickettsiaceae	*Rickettsia*
OTU_280	2.7659	0.8043	0.002	0.0218	Lactobacillales	Streptococcaceae	*Streptococcus*
OTU_45	2.9869	0.8524	0.0017	0.0207	Bacillales	Staphylococcaceae	*Staphylococcus*
OTU_235	5.7617	1.6456	0.0017	0.0207	Bacillales	Bacillaceae	*Bacillus*
OTU_104	7.9446	1.4446	<0.001	0.0005	Flavobacteriales	Flavobacteriaceae	Unassigned
OTU_119	24.4801	0.2463	<0.001	<0.001	Bacteroidales	Porphyromonadaceae	*Dysgonomonas*

*Note*

LME: linear mixed‐effect model; OTUs: operational taxonomic units; PHA: phytohaemagglutinin.

On the contrary, intensity of PHA response tended to increase with increasing abundance of OTUs from genus *Bacillus, Staphylococcus* (putatively *Staphylococcus saprophyticus*; 100% identity according to blastn searches), *Dysgonomonas* and *Streptococcus*. Bacteria from genus *Bacillus* include many common gut symbionts of vertebrates. On the other hand, *S. saprophyticus* is opportunistic pathogen causing inflammatory diseases of urinary tract in humans (Hovelius & Mårdh, 1984) and *Dysgonomonas* can cause gut inflammation in immunocompromised human subjects (Bangsborg, Frederiksen, & Bruun, 1990). Both these OTUs were previously detected in bird GM (Kropáčková, Pechmanová, et al., 2017; Kropáčková, Těšický, et al., 2017; Xenoulis et al., 2010). However, their effect on physiology and health of avian are still unknown. Many *Streptococcus* species are vertebrate commensal, but some represent opportunistic pathogens of various vertebrate taxa including birds (Benskin, Wilson, Jones, & Hartley, 2009). Unfortunately, our data did not allow reliable species‐level assignation of this particular OTU.

The contrasting effect of *Rickettsia* and *Staphylococcus* OTUs on PHA response suggests that putative bacterial pathogens can be associated both with attenuation and enhancement of PHA response. A similar contradictory pattern has been observed in the case of putative commensal or beneficial bacteria, with *Lactococcus* OTU abundance, in particular, being negatively related to PHA swelling and *Bacillus* sp. being positively related. We propose that these seemingly contrasting results are related to both interaction complexity between bacteria and the vertebrate immune system and to the complex immunological background of the PHA swelling response. Further studies targeting specific components of the bird immune system are required, therefore, in order to obtain a better understanding of how (a) host immune system interacts with GM and (b) how the overall pattern of such interactions differ from well‐established mammalian models.

## CONFLICT OF INTEREST

None declared.

## AUTHOR CONTRIBUTIONS

TA and JK designed the study. AP, MA, TA, RM, and OT carried out field sampling. LS, J‐FM, and RM performed laboratory analysis. JK and LS performed data analysis. TA, LS, and JK secured funding. JK drafted the manuscript. All authors provided helpful comments and recommendations and approved the final version of the manuscript.

## DATA ACCESSIBILITY

FASTQ files for individual samples are available at European Nucleotide Archive (project accession number: PRJEB27618, http://www.ebi.ac.uk/ena/data/view/PRJEB27618).

## References

[ece34454-bib-0001] Bangsborg, J. M. , Frederiksen, W. , & Bruun, B. (1990). Dysgonic fermenter 3‐associated abscess in a diabetic patient. Journal of Infection, 20, 237–240. 2341734 10.1016/0163-4453(90)91194-i

[ece34454-bib-0002] Bates, D. , Mächler, M. , Bolker, B. , & Walker, S. (2015). Fitting linear mixed‐effects models using lme4. Journal of Statistical Software, 67, 1–48. https://doi.org/10.18637/jss.v067.i01

[ece34454-bib-0003] Belkaid, Y. , & Hand, T. (2014). Role of the microbiota in immunity and inflammation. Cell, 157, 121–141. 10.1016/j.cell.2014.03.011 24679531 PMC4056765

[ece34454-bib-0004] Benskin, C. M. H. , Wilson, K. , Jones, K. , & Hartley, I. R. (2009). Bacterial pathogens in wild birds: A review of the frequency and effects of infection. Biological Reviews of the Cambridge Philosophical Society, 84, 349–373. 10.1111/j.1469-185X.2008.00076.x 19438430

[ece34454-bib-0005] Benson, A. K. , Kelly, S. A. , Legge, R. , Ma, F. , Low, S. J. , Kim, J. , … Pomp, D. (2010). Individuality in gut microbiota composition is a complex polygenic trait shaped by multiple environmental and host genetic factors. Proceedings of the National Academy of Sciences of the United States of America, 107, 18933–18938. 10.1073/pnas.1007028107 20937875 PMC2973891

[ece34454-bib-0006] Bolnick, D. I. , Snowberg, L. K. , Caporaso, J. G. , Lauber, C. , Knight, R. , & Stutz, W. E. (2014). Major Histocompatibility Complex class IIb polymorphism influences gut microbiota composition and diversity. Molecular Ecology, 23, 4831–4845. 10.1111/mec.12846 24975397

[ece34454-bib-0007] Bourgon, R. , Gentleman, R. , & Huber, W. (2010). Independent filtering increases detection power for high‐throughput experiments. Proceedings of the National Academy of Sciences of the United States of America, 107, 9546–9551. 10.1073/pnas.0914005107 20460310 PMC2906865

[ece34454-bib-0008] Bowers, E. K. , Hodges, C. J. , Forsman, A. M. , Vogel, L. A. , Masters, B. S. , Johnson, B. G. P. , … Sakaluk, S. K. (2014). Neonatal body condition, immune responsiveness, and hematocrit predict longevity in a wild bird population. Ecology, 95, 3027–3034. 10.1890/14-0418.1 25505800 PMC4260523

[ece34454-bib-0009] Boysen, P. , Eide, D. M. , & Storset, A. K. (2011). Natural killer cells in free‐living Mus musculus have a primed phenotype. Molecular Ecology, 20, 5103–5110. 10.1111/j.1365-294X.2011.05269.x 21895821

[ece34454-bib-0010] Caporaso, J. G. , Bittinger, K. , Bushman, F. D. , DeSantis, T. Z. , Andersen, G. L. , & Knight, R. (2010). PyNAST: A flexible tool for aligning sequences to a template alignment. Bioinformatics, 26, 266–267. 10.1093/bioinformatics/btp636 19914921 PMC2804299

[ece34454-bib-0011] Chang, P. V. , Hao, L. , Offermanns, S. , & Medzhitov, R. (2014). The microbial metabolite butyrate regulates intestinal macrophage function via histone deacetylase inhibition. Proceedings of the National Academy of Sciences of the United States of America, 111, 2247–2252. 10.1073/pnas.1322269111 24390544 PMC3926023

[ece34454-bib-0012] Cramp, S. , & Perrins, C. M. (Eds.). (1993). The birds of the Western Palearctic volume VII. Oxford, UK and New York, NY: Oxford University Press.

[ece34454-bib-0013] DeSantis, T. Z. , Hugenholtz, P. , Larsen, N. , Rojas, M. , Brodie, E. L. , Keller, K. , … Andersen, G. L. (2006). Greengenes, a chimera‐checked 16S rRNA gene database and workbench compatible with ARB. Applied and Environment Microbiology, 72, 5069–5072. 10.1128/AEM.03006-05 PMC1489311 16820507

[ece34454-bib-0014] Edgar, R. C. (2013). UPARSE: Highly accurate OTU sequences from microbial amplicon reads. Nature Methods, 10, 996–998. 10.1038/nmeth.2604 23955772

[ece34454-bib-0015] Fisher, K. , & Phillips, C. (2009). The ecology, epidemiology and virulence of Enterococcus. Microbiology, 155, 1749–1757. 10.1099/mic.0.026385-0 19383684

[ece34454-bib-0016] Flies, A. S. , Mansfield, L. S. , Grant, C. K. , Weldele, M. L. , & Holekamp, K. E. (2015). Markedly elevated antibody responses in wild versus captive spotted hyenas show that environmental and ecological factors are important modulators of immunity. PLoS ONE, 10, e0137679 10.1371/journal.pone.0137679 26444876 PMC4621877

[ece34454-bib-0017] González‐Braojos, S. , Vela, A. I. , Ruiz‐de‐Castañeda, R. , Briones, V. , & Moreno, J. (2012). Age‐related changes in abundance of enterococci and Enterobacteriaceae in Pied Flycatcher (*Ficedula hypoleuca*) nestlings and their association with growth. Journal of Ornithology, 153, 181–188. 10.1007/s10336-011-0725-y

[ece34454-bib-0018] Goto, N. , Kodama, H. , Okada, K. , & Fujimoto, Y. (1978). Suppression of phytohemagglutinin skin response in thymectomized chickens. Poultry Science, 57, 246–250. 10.3382/ps.0570246 674011

[ece34454-bib-0019] Han, K. J. , Lee, N.‐K. , Park, H. , & Paik, H.‐D. (2015). Anticancer and anti‐inflammatory activity of probiotic *Lactococcus lactis* NK34. Journal of Microbiology and Biotechnology, 25, 1697–1701. 10.4014/jmb.1503.03033 26165315

[ece34454-bib-0020] Hawley, D. M. , Sydenstricker, K. V. , Kollias, G. V. , & Dhondt, A. A. (2005). Genetic diversity predicts pathogen resistance and cell‐mediated immunocompetence in house finches. Biology Letters, 1, 326–329. 10.1098/rsbl.2005.0303 17148199 PMC1617150

[ece34454-bib-0021] Hildebrand, F. , Tadeo, R. , Voigt, A. Y. , Bork, P. , & Raes, J. (2014). LotuS: An efficient and user‐friendly OTU processing pipeline. Microbiome, 2, 30 10.1186/2049-2618-2-30 27367037 PMC4179863

[ece34454-bib-0022] Hird, S. M. , Carstens, B. C. , Cardiff, S. W. , Dittmann, D. L. , & Brumfield, R. T. (2014). Sampling locality is more detectable than taxonomy or ecology in the gut microbiota of the brood‐parasitic Brown‐headed Cowbird (*Molothrus ater*). PeerJ, 2, e321 10.7717/peerj.321 24711971 PMC3970801

[ece34454-bib-0023] Hovelius, B. , & Mårdh, P.‐A. (1984). *Staphylococcus saprophyticus* as a common cause of urinary tract infections. Clinical Infectious Diseases, 6, 328–337. 10.1093/clinids/6.3.328 6377440

[ece34454-bib-0024] Ivanov, I. I. , Atarashi, K. , Manel, N. , Brodie, E. L. , Shima, T. , Karaoz, U. , … Littman, D. R. (2009). Induction of intestinal Th17 cells by segmented filamentous bacteria. Cell, 139, 485–498. 10.1016/j.cell.2009.09.033 19836068 PMC2796826

[ece34454-bib-0025] Jones, R. M. , Luo, L. , Ardita, C. S. , Richardson, A. N. , Kwon, Y. M. , Mercante, J. W. , … Neish, A. S. (2013). Symbiotic lactobacilli stimulate gut epithelial proliferation via Nox‐mediated generation of reactive oxygen species. The EMBO Journal, 32, 3017–3028. 10.1038/emboj.2013.224 24141879 PMC3844951

[ece34454-bib-0026] Jumpertz, R. , Le, D. S. , Turnbaugh, P. J. , Trinidad, C. , Bogardus, C. , Gordon, J. I. , & Krakoff, J. (2011). Energy‐balance studies reveal associations between gut microbes, caloric load, and nutrient absorption in humans. American Journal of Clinical Nutrition, 94, 58–65. 10.3945/ajcn.110.010132 21543530 PMC3127503

[ece34454-bib-0027] Kim, C. H. , Park, J. , & Kim, M. (2014). Gut microbiota‐derived short‐chain fatty acids, T cells, and inflammation. Immune Network, 14, 277–288. 10.4110/in.2014.14.6.277 25550694 PMC4275385

[ece34454-bib-0028] Klindworth, A. , Pruesse, E. , Schweer, T. , Peplies, J. , Quast, C. , Horn, M. , & Glöckner, F. O. (2013). Evaluation of general 16S ribosomal RNA gene PCR primers for classical and next‐generation sequencing‐based diversity studies. Nucleic Acids Research, 41, e1 10.1093/nar/gks808 22933715 PMC3592464

[ece34454-bib-0029] Kreisinger, J. , Čížková, D. , Vohánka, J. , & Piálek, J. (2014). Gastrointestinal microbiota of wild and inbred individuals of two house mouse subspecies assessed using high‐throughput parallel pyrosequencing. Molecular Ecology, 23, 5048–5060. 10.1111/mec.12909 25204516

[ece34454-bib-0030] Kreisinger, J. , Kropáčková, L. , Petrželková, A. , Adámková, M. , Tomášek, O. , Martin, J.‐F. , … Albrecht, T. (2017). Temporal stability and the effect of transgenerational transfer on fecal microbiota structure in a long distance migratory bird. Frontiers in Microbiology, 8, 50 10.3389/fmicb.2017.00050 28220109 PMC5292904

[ece34454-bib-0031] Kropáčková, L. , Pechmanová, H. , Vinkler, M. , Svobodová, J. , Velová, H. , Těšičký, M. , … Kreisinger, J. (2017). Variation between the oral and faecal microbiota in a free‐living passerine bird, the great tit (*Parus major*). PLoS ONE, 12, e0179945 10.1371/journal.pone.0179945 28662106 PMC5491070

[ece34454-bib-0032] Kropáčková, L. , Těšický, M. , Albrecht, T. , Kubovčiak, J. , Čížková, D. , Tomášek, O. , … Kreisinger, J. (2017). Co‐diversification of gastrointestinal microbiota and phylogeny in passerines is not explained by ecological divergence. Molecular Ecology, 26, 5292–5304. 10.1111/mec.14144 28401612

[ece34454-bib-0033] Kurtz, J. , Kalbe, M. , Aeschlimann, P. B. , Häberli, M. A. , Wegner, K. M. , Reusch, T. B. H. , & Milinski, M. (2004). Major histocompatibility complex diversity influences parasite resistance and innate immunity in sticklebacks. Proceedings of the Royal Society of London B: Biological Sciences, 271, 197–204. 10.1098/rspb.2003.2567 PMC1691569 15058398

[ece34454-bib-0034] Legendre, P. , & Anderson, M. J. (1999). Distance‐based redundancy analysis: Testing multispecies responses in multifactorial ecological experiments. Ecological Monographs, 69, 1–24. 10.2307/2657192

[ece34454-bib-0035] Ley, R. E. , Hamady, M. , Lozupone, C. , Turnbaugh, P. , Ramey, R. R. , Bircher, J. S. , … Gordon, J. I. (2008). Evolution of mammals and their gut microbes. Science, 320, 1647–1651. 10.1126/science.1155725 18497261 PMC2649005

[ece34454-bib-0036] Lifjeld, J. T. , Dunn, P. O. , & Whittingham, L. A. (2002). Short‐term fluctuations in cellular immunity of tree swallows feeding nestlings. Oecologia, 130, 185–190. 10.1007/s004420100798 28547140

[ece34454-bib-0037] Love, M. I. , Huber, W. , & Anders, S. (2014). Moderated estimation of fold change and dispersion for RNA‐seq data with DESeq2. Genome Biology, 15, 550 10.1186/s13059-014-0550-8 25516281 PMC4302049

[ece34454-bib-0038] Lozupone, C. , & Knight, R. (2005). UniFrac: A new phylogenetic method for comparing microbial communities. Applied and Environment Microbiology, 71, 8228–8235. 10.1128/AEM.71.12.8228-8235.2005 PMC1317376 16332807

[ece34454-bib-0039] Luerce, T. D. , Gomes‐Santos, A. C. , Rocha, C. S. , Moreira, T. G. , Cruz, D. N. , Lemos, L. , … Miyoshi, A. (2014). Anti‐inflammatory effects of *Lactococcus lactis* NCDO 2118 during the remission period of chemically induced colitis. Gut Pathogens, 6, 33 10.1186/1757-4749-6-33 25110521 PMC4126083

[ece34454-bib-0040] Madsen, T. , & Ujvari, B. (2006). MHC class I variation associates with parasite resistance and longevity in tropical pythons. Journal of Evolutionary Biology, 19, 1973–1978. 10.1111/j.1420-9101.2006.01158.x 17040395

[ece34454-bib-0041] Maizels, R. M. , & Nussey, D. H. (2013). Into the wild: Digging at immunology's evolutionary roots. Nature Immunology, 14, 879–883. 10.1038/ni.2643 23959175

[ece34454-bib-0042] McKenzie, V. J. , Song, S. J. , Delsuc, F. , Prest, T. L. , Oliverio, A. M. , Korpita, T. M. , … Knight, R. (2017). The effects of captivity on the mammalian gut microbiome. Integrative and Comparative Biology, 57, 690–704. 10.1093/icb/icx090 28985326 PMC5978021

[ece34454-bib-0043] McMurdie, P. J. , & Holmes, S. (2013). phyloseq: An R package for reproducible interactive analysis and graphics of microbiome census data. PLoS ONE, 8, e61217 10.1371/journal.pone.0061217 23630581 PMC3632530

[ece34454-bib-0044] Merlo, J. L. , Cutrera, A. P. , & Zenuto, R. R. (2016). Parasite infection negatively affects PHA‐triggered inflammation in the subterranean rodent *Ctenomys talarum* . Journal of Experimental Zoology, 325, 132–141. 10.1002/jez.2003 26718121

[ece34454-bib-0045] Møller, A. P. (2001). The effect of dairy farming on barn swallow *Hirundo rustica* abundance, distribution and reproduction. Journal of Applied Ecology, 38, 378–389. 10.1046/j.1365-2664.2001.00593.x

[ece34454-bib-0046] Møller, A. P. , & Petrie, M. (2002). Condition dependence, multiple sexual signals, and immunocompetence in peacocks. Behavioral Ecology, 13, 248–253. 10.1093/beheco/13.2.248

[ece34454-bib-0047] Oksanen, J. , Blanchet, F. G. , Kindt, R. , Legendre, P. , Minchin, P. R. , O'Hara, R. B. , … Wagner, H. (2013). vegan: Community ecology package. available at: https://cran.ism.ac.jp/web/packages/vegan

[ece34454-bib-0048] Ondov, B. D. , Bergman, N. H. , & Phillippy, A. M. (2011). Interactive metagenomic visualization in a Web browser. BMC Bioinformatics, 12, 385 10.1186/1471-2105-12-385 21961884 PMC3190407

[ece34454-bib-0049] Parola, P. , & Didier, R. (2001). Ticks and tickborne bacterial diseases in humans: An emerging infectious threat. Clinical Infectious Diseases, 32, 897–928. 10.1086/319347 11247714

[ece34454-bib-0050] Petrželková, A. , Michálková, R. , Albrechtová, J. , Cepák, J. , Honza, M. , Kreisinger, J. , … Albrecht, T. (2015). Brood parasitism and quasi‐parasitism in the European barn swallow (*Hirundo rustica rustica*). Behavioral Ecology and Sociobiology, 69, 1405–1414. 10.1007/s00265-015-1953-6

[ece34454-bib-0051] Price, M. N. , Dehal, P. S. , & Arkin, A. P. (2010). FastTree 2—Approximately maximum‐likelihood trees for large alignments. PLoS ONE, 5, e9490 10.1371/journal.pone.0009490 20224823 PMC2835736

[ece34454-bib-0052] Qin, J. , Li, R. , Raes, J. , Arumugam, M. , Burgdorf, K. S. , Manichanh, C. , … Wang, J. (2010). A human gut microbial gene catalogue established by metagenomic sequencing. Nature, 464, 59–65. 10.1038/nature08821 20203603 PMC3779803

[ece34454-bib-0053] R Core Team (2016). R: A language and environment for statistical computing. Vienna, Austria: R Foundation for Statistical Computing Retrieved from https://www.R-project.org/

[ece34454-bib-0054] Ruiz‐Rodríguez, M. , Soler, J. J. , Lucas, F. S. , Heeb, P. , José Palacios, M. , Martín‐Gálvez, D. , … Soler, M. (2009). Bacterial diversity at the cloaca relates to an immune response in magpie *Pica pica* and to body condition of great spotted cuckoo *Clamator glandarius* nestlings. Journal of Avian Biology, 40, 42–48. 10.1111/j.1600-048X.2008.04471.x

[ece34454-bib-0055] Saino, N. , Ambrosini, R. , Martinelli, R. , & Møller, A. P. (2002). Mate fidelity, senescence in breeding performance and reproductive trade‐offs in the barn swallow. Journal of Animal Ecology, 71, 309–319. 10.1046/j.1365-2656.2002.00600.x

[ece34454-bib-0056] Saino, N. , Ferrari, R. , Romano, M. , Martinelli, R. , & Møller, A. P. (2003). Experimental manipulation of egg carotenoids affects immunity of barn swallow nestlings. Proceedings of the Royal Society of London B: Biological Sciences, 270, 2485–2489. 10.1098/rspb.2003.2534 PMC1691538 14667340

[ece34454-bib-0057] Saks, L. , Karu, U. , Ots, I. , & Hõrak, P. (2006). Do standard measures of immunocompetence reflect parasite resistance? The case of greenfinch coccidiosis. Functional Ecology, 20, 75–82. 10.1111/j.1365-2435.2006.01068.x

[ece34454-bib-0058] Schloss, P. D. , Westcott, S. L. , Ryabin, T. , Hall, J. R. , Hartmann, M. , Hollister, E. B. , … Weber, C. F. (2009). Introducing mothur: Open‐source, platform‐independent, community‐supported software for describing and comparing microbial communities. Applied and Environment Microbiology, 75, 7537–7541. 10.1128/AEM.01541-09 PMC2786419 19801464

[ece34454-bib-0059] Sekirov, I. , Russell, S. L. , Antunes, L. C. M. , & Finlay, B. B. (2010). Gut microbiota in health and disease. Physiological Reviews, 90, 859–904. 10.1152/physrev.00045.2009 20664075

[ece34454-bib-0060] Sjögren, Y. M. , Tomicic, S. , Lundberg, A. , Böttcher, M. F. , Björkstén, B. , Sverremark‐Ekström, E. , & Jenmalm, M. C. (2009). Influence of early gut microbiota on the maturation of childhood mucosal and systemic immune responses. Clinical & Experimental Allergy, 39, 1842–1851. 10.1111/j.1365-2222.2009.03326.x 19735274

[ece34454-bib-0061] Sommer, S. (2005). The importance of immune gene variability (MHC) in evolutionary ecology and conservation. Frontiers in Zoology, 2, 16 10.1186/1742-9994-2-16 16242022 PMC1282567

[ece34454-bib-0062] Storey, J. D. , & Tibshirani, R. (2003). Statistical significance for genomewide studies. Proceedings of the National Academy of Sciences of the United States of America, 100, 9440–9445. 10.1073/pnas.1530509100 12883005 PMC170937

[ece34454-bib-0063] Sullam, K. E. , Essinger, S. D. , Lozupone, C. A. , O'Connor, M. P. , Rosen, G. L. , Knight, R. , … Russell, J. A. (2012). Environmental and ecological factors that shape the gut bacterial communities of fish: A meta‐analysis. Molecular Ecology, 21, 3363–3378. 10.1111/j.1365-294X.2012.05552.x 22486918 PMC3882143

[ece34454-bib-0064] Tella, J. L. , Lemus, J. A. , Carrete, M. , & Blanco, G. (2008). The PHA test reflects acquired T‐cell mediated immunocompetence in birds. PLoS ONE, 3, e3295 10.1371/journal.pone.0003295 18820730 PMC2546448

[ece34454-bib-0065] van der Most, P. J. , de Jong, B. , Parmentier, H. K. , & Verhulst, S. (2011). Trade‐off between growth and immune function: A meta‐analysis of selection experiments. Functional Ecology, 25, 74–80. 10.1111/j.1365-2435.2010.01800.x

[ece34454-bib-0066] Vatanen, T. , Kostic, A. D. , d'Hennezel, E. , Siljander, H. , Franzosa, E. A. , Yassour, M. , … Xavier, R. J. (2016). Variation in microbiome LPS immunogenicity contributes to autoimmunity in humans. Cell, 165, 842–853. 10.1016/j.cell.2016.04.007 27133167 PMC4950857

[ece34454-bib-0067] Vinkler, M. , Bainová, H. , & Albrecht, T. (2010). Functional analysis of the skin‐swelling response to phytohaemagglutinin. Functional Ecology, 24, 1081–1086. 10.1111/j.1365-2435.2010.01711.x

[ece34454-bib-0068] Vinkler, M. , Schnitzer, J. , Munclinger, P. , & Albrecht, T. (2012). Phytohaemagglutinin skin‐swelling test in scarlet rosefinch males: Low‐quality birds respond more strongly. Animal Behaviour, 83, 17–23. 10.1016/j.anbehav.2011.10.001

[ece34454-bib-0069] Waite, D. W. , & Taylor, M. W. (2014). Characterizing the avian gut microbiota: Membership, driving influences, and potential function. Frontiers in Microbiology, 5, 223 10.3389/fmicb.2014.00223 24904538 PMC4032936

[ece34454-bib-0070] Wu, H.‐J. , & Wu, E. (2012). The role of gut microbiota in immune homeostasis and autoimmunity. Gut Microbes, 3, 4–14. 10.4161/gmic.19320 22356853 PMC3337124

[ece34454-bib-0071] Xenoulis, P. G. , Gray, P. L. , Brightsmith, D. , Palculict, B. , Hoppes, S. , Steiner, J. M. , … Suchodolski, J. S. (2010). Molecular characterization of the cloacal microbiota of wild and captive parrots. Veterinary Microbiology, 146, 320–325. 10.1016/j.vetmic.2010.05.024 20646876

[ece34454-bib-0072] Zhang, J. , Kobert, K. , Flouri, T. , & Stamatakis, A. (2014). PEAR: A fast and accurate Illumina Paired‐End reAd mergeR. Bioinformatics, 30, 614–620. 10.1093/bioinformatics/btt593 24142950 PMC3933873

[ece34454-bib-0073] Zhang, X. , Mallick, H. , Tang, Z. , Zhang, L. , Cui, X. , Benson, A. K. , & Yi, N. (2017). Negative binomial mixed models for analyzing microbiome count data. BMC Bioinformatics, 18, 4 10.1186/s12859-016-1441-7 28049409 PMC5209949

